# P011 Vanishing bone of the skull: A rare case of Gorham-Stout Disease

**DOI:** 10.1093/rap/rkag113.041

**Published:** 2026-11-03

**Authors:** Selene Basso, Kassim Javaid, Luke Sammut

**Affiliations:** Queen Alexandra Hospital, Portsmouth, United Kingdom; Nuffield Department of Orthopaedics, Rheumatology and Muscoloskeletal Sciences, Oxford, United Kingdom; Queen Alexandra Hospital, Portsmouth, United Kingdom

## Abstract

**Introduction:**

Gorham-Stout disease (GSD), also known as vanishing bone disease, is a rare osteolytic disorder characterised by progressive proliferation of lymphatic vessels within the bone.

Isolated involvement of the calvaria is uncommon, with few cases reported in the literature. We describe a young man with this unusual presentation, highlighting the diagnostic challenges and the importance of multidisciplinary assessment.

**Case description:**

A man in his 30s presented with a hard, painless lump over the left parietal bone, first identified by his hairdresser and progressively enlarging over the preceding six months. He reported no systemic symptoms, was otherwise well, and baseline blood tests were normal. His past medical history included a hiatus hernia repair.

Head computed tomography (CT) demonstrated focal thickening of the left parietal bone with an intact cortex, prompting neurosurgical referral. Subsequent isotope bone scanning and head magnetic resonance imaging (MRI) suggested fibrous dysplasia. The case was reviewed at the neuro-oncology multi-disciplinary (MDT) meeting, where the imaging was considered atypical for fibrous dysplasia because of the associated bone loss, and therefore GSD was included in the differential diagnosis. Six-monthly MRI surveillance was initiated, and rheumatology input was sought as no neurosurgical intervention was indicated.

The patient was referred to the metabolic bone MDT in Oxford, who advised biopsy to identify lymphatic vessel proliferation within bone, required for confirmation of GSD. Neurosurgery performed the biopsy, and samples were submitted for histopathological and genetic analysis. He remained asymptomatic throughout and was advised to avoid contact sports due to cortical thinning of the skull.

The patient commenced monthly intravenous zoledronic acid with calcium supplementation for six months, to prevent further bone loss while awaiting definitive diagnostic clarification.

Repeat CT imaging demonstrated a stable 10-cm area of full-thickness thinning of the left parieto-occipital skull vault, with loss of the outer diploic plate into the diploic space and eggshell thinning of the inner plate posteriorly. Findings were unchanged from the previous year. The patient continues under follow-up with the metabolic bone team.

**Discussion:**

Gorham-Stout disease (GSD) remains a challenging condition to diagnose and treat due to its rarity, particularly when involving the calvaria.

This case highlights the diagnostic uncertainty that can arise, as the initial radiological appearances were more suggestive of fibrous dysplasia. Multidisciplinary review, including neuroradiology, neurosurgery, rheumatology and the metabolic bone team, was essential in establishing the diagnosis and guiding management. In the absence of established treatment guidelines or further surgical options, close radiological surveillance and a six-month course of intravenous bisphosphonate therapy were used to monitor and potentially limit disease progression.

**Key learning points:**

• This case highlights the diagnostic challenge of Gorham–Stout disease, which initially appeared more consistent with fibrous dysplasia on imaging. Early multidisciplinary involvement was crucial in revisiting the working diagnosis and guiding further investigation and management.

